# Mitigating the effects of reference sequence bias in single-multiplex massively parallel sequencing of the mitochondrial DNA control region

**DOI:** 10.1016/j.fsigen.2019.01.008

**Published:** 2019-05

**Authors:** Tunde I. Huszar, Jon H. Wetton, Mark A. Jobling

**Affiliations:** Department of Genetics & Genome Biology, University of Leicester, University Road, Leicester LE1 7RH UK

**Keywords:** mtDNA, Control region, Massively parallel sequencing (MPS), Heteroplasmy, Single nucleotide polymorphism (SNP), PowerSeq™ CRM Nested System, Overarching Read Enrichment Option (OREO)

## Abstract

Sequence analysis of the mitochondrial DNA (mtDNA) control region can provide forensically useful information, particularly in challenging samples where autosomal DNA profiling fails. Sub-division of the 1122-bp region into shorter PCR fragments improves data recovery, and such fragments can be analysed together via massively parallel sequencing (MPS). Here, we generate mtDNA data using the prototype PowerSeq™ Auto/Mito/Y System (Promega) MPS assay, in which a single PCR reaction amplifies ten overlapping amplicons of the control region, in a set of 101 highly diverse samples representing most major clades of the mtDNA phylogeny. The overlapping multiplex design leads to non-uniform coverage in the regions of overlap, where it is further increased by short amplicons generated alongside the intended products. Primer sequences in targeted amplification libraries are a potential source of reference sequence bias and thus should be removed, but the proprietary nature of the primers in commercial kits necessitates an alternative approach that minimises data loss: here, we introduce the bioinformatic selection of sequencing reads spanning putative primer sites (Overarching Read Enrichment Option, OREO). While OREO performs well in mitigating the effects of primer sequences at the ends of sequence reads, we still find evidence of the internalisation of primer-derived sequences by overlap extension, which may compromise the ability to call variants or to measure heteroplasmy in primer-binding regions. The commercially available PowerSeq™ CRM Nested System design prevents primer internalisation, as shown in a reanalysis of a subset of 57 samples that contain possible heteroplasmies. In combination with OREO, the CRM Nested kit mitigates reference sequence bias, allowing heteroplasmic variants to be estimated down to a 5% threshold. Provided appropriate steps are taken in data processing, single-reaction multiplex assays represent robust tools to analyse mtDNA control region variation. The OREO approach will allow users to bypass the effects of unknown primer sequences in any single-reaction tiled multiplex and eliminate primer-derived bias in overlapping amplicon sequencing studies, in both forensic and non-forensic settings.

## Highlights

• mtDNA control region of 101 diverse samples amplified in a single reaction as 10 overlapping amplicons and sequenced via MPS.
• Primers create reference bias, compromising ability to call variants or heteroplasmy in primer-binding regions.
• Bioinformatic selection of overarching reads bypasses effects of proprietary primers and mitigates bias.
• Data processing permits accurate calling of variants, and heteroplasmies down to 5% level.

## 1 Introduction

When standard autosomal STR profiling fails because DNA in a biological sample is limited or highly degraded, forensically useful information can often be obtained via analysis of mitochondrial DNA (mtDNA) [1], thanks to its relatively high copy number in cells compared to the nuclear genome. Mitochondrial DNA is also the only viable source of genetic evidence from samples such as hair shafts [2], in which nuclear DNA is naturally depleted. Forensic practice has largely focused on analysing the 1122-bp control region, in which high levels of variation can be detected [3,4], and which can be sequenced via Sanger technology from a single PCR amplicon when sample quality allows.

In particularly poor-quality samples, a set of overlapping shorter PCR amplicons spanning the control region can be used to generate sequence data; this was the approach taken in early ancient DNA analyses [5,6]. However, the variability of the control region that makes it useful also makes PCR primer design challenging, because sequence variants within primer binding sites could inhibit the amplification due to mismatching [3,7]. Surveying known variation in large reference mtDNA datasets has allowed rational primer design that avoids the most mutable sites [7]. However, the multiple-amplicon approach to control region analysis remains vulnerable to variants within particular mtDNA haplogroups that may be unaccounted for in the primer design, and therefore could inhibit amplification in an amplicon- and sample-specific way in casework. If amplification of the primary source’s mtDNA is reduced due to such mismatches, a non-mismatched minor source or nuclear mitochondrial DNA (numt) sequence might be amplified relatively efficiently, leading to complications of interpretation.

**Fig. 1:**
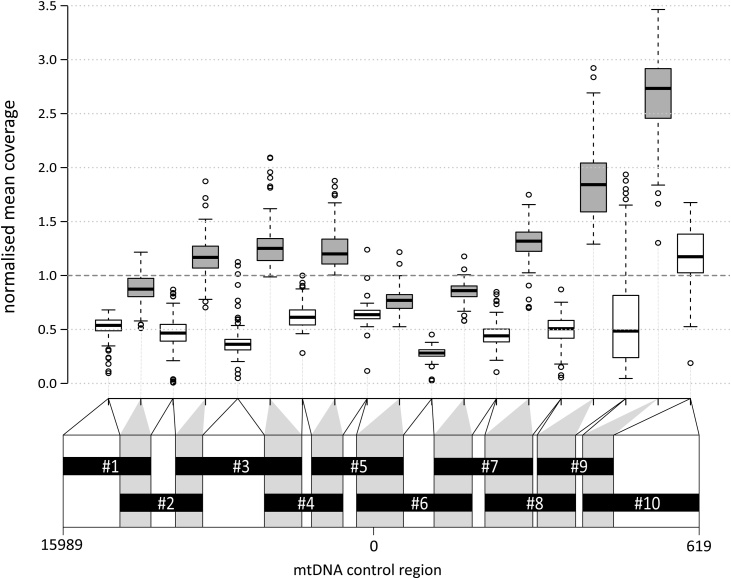
Schematic representation of the multiplex assay design, and non-uniform coverage over the control region observed in the prototype kit. The lower part shows the ten designed amplicons and their overlapping (grey) and non-overlapping (white) segments. Positions and sizes are approximately to scale. The boxplot above shows mean coverage for all 101 samples analysed, for each segment, normalised to sample means. Centre lines indicate the medians; box limits are the 25th and 75th percentiles; whiskers extend 1.5 times the interquartile range from the 25th and 75th percentiles, outliers are represented by circles. Note the relatively high coverage for overlapping segments (grey).

Amplicons generated from within the control region (Fig. 1; between 144 and 237 bp in length [7]) have traditionally been analysed individually by Sanger sequencing [7,8]. However, the process can be made more efficient by multiplexing followed by massively parallel sequencing (MPS) [9]. Different commercial assay designs exist, either as two multiplexes, each containing alternate amplicons (e.g. Precision ID mtDNA Control Region Panel; Thermo Fisher - not tested in this study), or as a single multiplex (Fig. 1). The latter has the advantage of simplifying workflow and reducing sample usage. However, it also has the potential disadvantage that primer pairs will act in combinations other than those intended in the design and create many additional products that may be preferentially amplified because of their smaller size. In an MPS setting, such products consume sequencing capacity without contributing additional information about the mtDNA template and are expected to further increase the non-uniform coverage across the control region.

A further potential problem of targeted amplification of the control region is the introduction of reference sequence bias into the reads. Primers are generally designed to match the reference sequence, therefore in a sample carrying an unexpected alternative variant the sequence reads could present a mixture of alternative alleles originating from the actual mtDNA, and reference alleles originating from the primer sequences themselves at a variant position. If the primer sequences are known, these can be removed during data processing by trimming, but in commercial kits this exact information is proprietary, and therefore unavailable. If reads remain untrimmed of primer sequences, the preferentially amplified short amplicons (here 52 bp to 91 bp) in a single-reaction design further enhance the reference sequence bias due to their large contribution to the coverage, and to the fact that most of their content is primer-derived, and hence reference-biased. This bias is also expected to influence the accurate assessment of heteroplasmy - the presence of more than one control region sequence type due to mutation *in vivo* [4]. If heteroplasmic variants lie within primer-binding sites, the level at which these are detected might be underestimated, because they are diluted by the presence of reads with primer-derived sequences. Under the reasonable assumption that commercial designs use degenerate primer sets to account for common variants that lie under the primer-binding sites, these alternative primers, even when incorporated with lower efficiency, could introduce false low-level variants not intrinsic to the samples at the affected positions. A method is therefore required either during the library preparation (e.g. partial digestion of primers off the generated amplicons [10] used in the Precision ID mtDNA Control Region Panel; Thermo Fisher) or during data processing, that accounts for the presence of the proprietary primer-derived sequences and minimises the effect of these on the data prior to reporting.

Here, we generate data using a prototype single-reaction mtDNA MPS multiplex across a highly diverse set of 101 DNA samples that cover most of the major clades of the mitochondrial DNA haplogroup tree. We also test a subset of the same samples with the commercially available PowerSeq™ CRM Nested System in order to assess its improved accuracy in detecting variants and heteroplasmy in the regions affected in the prototype by primer-derived bias. We develop bioinformatic methods to decrease the effects of non-uniform coverage and reference sequence bias in both systems, thereby improving the detection of variants, and the quantification of heteroplasmy.

## 2 Materials and methods

### 2.1 DNA samples

One hundred male DNA samples were selected from a previously described set of 448 [11]. Sample details are given in Table S1. Quantities of double-stranded DNA were verified prior to PCR using a Qubit® 2.0 fluorometer (Thermo Fisher Scientific) with the Qubit® dsDNA HS kit. In addition, to control for operator contamination, DNA was extracted from a buccal swab of the operator (TIH) using the DNA IQ^™^ System (Promega), giving a total of 101 analysed DNA samples.

### 2.2 PCR amplification and sequencing

A segment of mitochondrial DNA (from position 15,989 to 619, which includes the control region [from 16,024 to 576]) was amplified in a single reaction generating ten overlapping amplicons from 0.5 ng template DNA using the prototype multiplex PowerSeq™ Auto/Mito/Y System (Promega), following the manufacturer’s recommended protocol. Results obtained for the Y-STRs have been published previously [12], and results for autosomal markers will be described elsewhere. In this paper, when we refer to a ‘single-reaction multiplex’, we consider the mtDNA component of the Auto/Mito/Y System only.

Library preparation and sequencing on a MiSeq® FGx (Illumina) sequencer were performed as described previously [12].

A subset of 57 samples that showed potential heteroplasmy were subsequently analysed using the commercially available PowerSeq™ CRM Nested System to assess its ability to detect heteroplasmic sites more accurately than the prototype version. The library preparation of these samples followed the manufacturer’s recommendations including the quantitation of the prepared libraries with the PowerSeq™ Quant MS System (Promega) and sequencing was carried out on a MiSeq® FGx (Illumina) sequencer in “research use only” (RUO) mode via the “Generate FASTQ” workflow with “FASTQ Only” application and paired-end (PE) method using the MiSeq®v3 (600 cycles) reagent kit.

### 2.3 Data processing and analyses

Quality-checked FASTQ files were generated as described previously [12]. Variants were called using a standard variant calling pipeline, adjusted to amplicon sequencing (omitting the duplicate removal step). The pipeline used the tools BWA v0.7.12 [13] for alignment, SAMtools v1.3.1 [14] for generating, sorting and indexing BAM files and calling variants, Picard v2.1.0 (http://broadinstitute.github.io/picard) for adding readgroups, GATK v3.4-0 [15] for local realignment, base calibration and coverage calculations, and VCFtools v0.1.15 [16] for filtering variants. The reads were aligned to the revised Cambridge Reference Sequence ([17] rCRS, GenBank accession: NC_012920.1). Variants were also visualised and confirmed using the Integrative Genomics Viewer (IGV) tool [18].

To seek the presence of sequence reads amplified from numt sequences, in-house Bash scripts were used, designed to select reads by focusing on numt-specific variants. To attempt assessments of the level of heteroplasmy and to interpret insertions and deletions and other variants found in ambiguous regions, such as homopolymeric tracts and the AC-repeat region, in-house Bash scripts were also used. To increase the confidence of lower-level heteroplasmy detection and quantitation within the putative primer-binding regions we applied the ‘Overarching Read Enrichment Option’ (OREO), described in the main text. We also tested two tools designed for the analysis of mtDNA MPS data: mtDNA-Server [19]; and the trial version of the commercial software GeneMarkerHTS v1.2.2.1338 (SoftGenetics, LLC).

Three methods were tested to account for unknown primer sequences: Cutadapt [20] and seqtk (available from: https://github.com/lh3/seqtk) to trim reads, and BAMClipper [21] to trim BAM files. For the analysis of the commercially available PowerSeq™ CRM Nested System data we also used Trimmomatic v0.32 software [22] for *in silico* size-selection as an additional step incorporated into data processing.

Validation of the results was performed by comparing SNP calls against a total of 65/101 independently sequenced samples, as follows: 58 from previously published data [23], of which 23 were also sequenced by the 1000 Genomes Project [24], thereby providing a three-way comparison; six additional 1000 Genomes Project samples; and one operator (TIH) control sample sequenced commercially (GenBank accession: MG551929).

Called variants were checked for correct nomenclature in a phylogenetic alignment context using the SAM2 tool of the EMPOP mtDNA database, v4/R11 [25]. Haplogroups were predicted and their relationships visualised using HaploGrep2 [26]. A maximum-likelihood phylogenetic tree of the control region (from position 15,989 to 619) was constructed using MEGA v6.06 [27] then visualised using FigTree v1.4.3 [28].

Graphs and diagrams were created using Microsoft Office and R software [29].

## 3 Results

We analysed a set of DNA samples that were selected previously to establish a phylogenetic framework for maximum diversity of the male-specific region of the Y chromosome [12]. These samples derive from ethnically diverse individuals including Europeans, Asians and Africans (Table S1).

We used Promega’s prototype PowerSeq™ Auto/Mito/Y System to generate MPS data from the mitochondrial control region of each of the 101 samples. The mtDNA-specific components of this kit amplify ten overlapping PCR fragments in the size range 144–237 bp that cover the control region as shown in Fig. 1. As expected given the design, coverage across the region is non-uniform: where the designed amplicons overlap (‘overlapping segments’) both amplicons contribute to elevated coverage; this imbalance may increase further by short PCR products equivalent to the length of the overlap itself. To reflect this, coverage and read statistics are calculated per segment as shown in Fig. 1, and as described in Table S2. With the analytical threshold set to 20 × coverage, for 24-plex and 96-plex sample pooling during library preparations we observed a mean of ˜17,700 × and ˜5500 × sequence coverage over the control region, with lowest values for non-overlapping segments of 1640 ×, and 57 × respectively. The coverage range observed in the prototype kit for mtDNA-derived reads was suitable for calling SNPs or indels, and allowed the evaluation of heteroplasmic sites down to a conservative level of 10%. At heteroplasmic sites the minor component was also required to meet the 20 × minimum read depth criterion (Fig. S2).

### 3.1 Calling variants in the control region

We attempted to call variants using standard approaches (Materials & methods), applying a 5% detection threshold and an initial calling threshold of 10% (comparable to the approximate Sanger sequencing threshold at which homoplasmic and heteroplasmic calls can be distinguished). To define these thresholds, we considered background error levels and coverage values (Fig. S2).

Considering that ten primer pairs are used to amplify a 1.2-kb segment, at least one third of the amplified region may be affected by primer-derived sequences. In order to reduce potential bias introduced by primers [21,30] at the ends of reads during variant detection, we tried different conventional approaches to trimming (See *Data processing and analyses* section of *Materials and methods*). We attempted to remove 20-26-nucleotide sequences from the read ends without creating coverage gaps, but nonetheless detected over 100 cases of apparent heteroplasmy in the samples. This is unexpectedly high compared with available data [31], therefore we suspected that our blind trimming efforts without the knowledge of exact primer sequences were still unsuccessful and that heteroplasmy levels were being inflated via the persistence of the unknown primer sequences in the data. Since primers are generally designed based on the reference sequence, this mainly constitutes ‘reference sequence bias’, but it is also possible that degenerate primers could contribute to the detection of low-level variants.

**Fig. 2:**
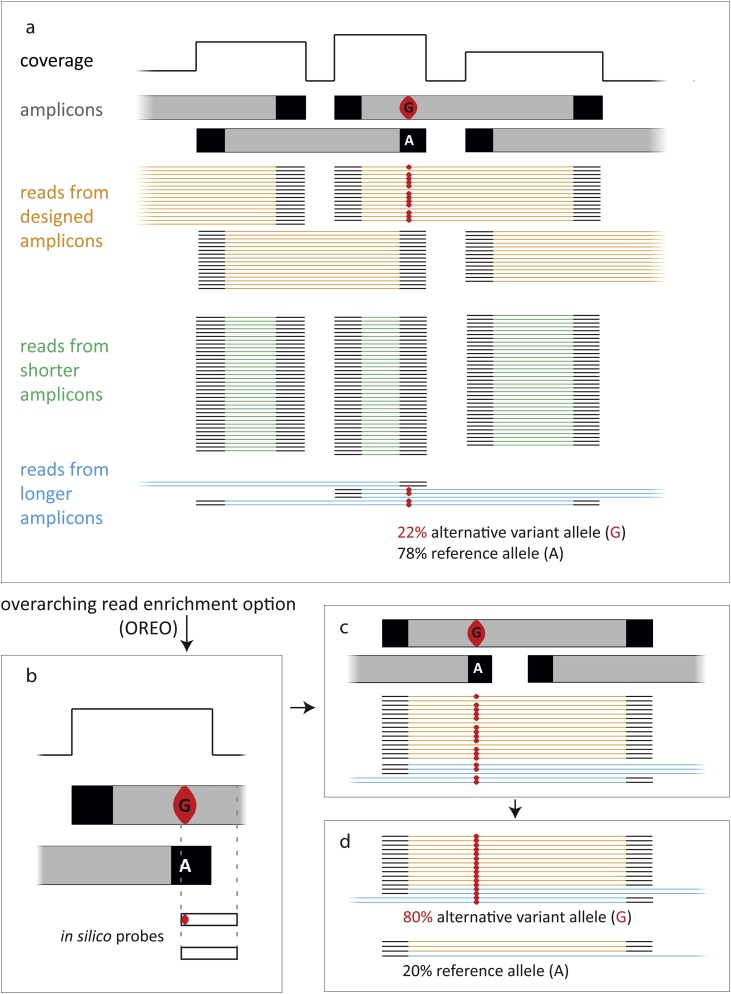
Improving estimation of heteroplasmy using Overarching Read Enrichment Option (OREO). a) When overlapping amplicons (grey bars, top) are used to generate sequence data from the control region an alternative data processing approach is required because exact primer-derived sequences (black boxes) are not known. The sequence reads derive from the designed amplicons (orange), short overlapping amplicons (green) and longer reads (blue). b) The overarching read enrichment option (OREO) filters reads for the presence of specific *in silico* ‘probe’ sequences (white boxes). The probes are designed to enrich for reads spanning the primer site, thus excluding reads that carry variants copied from the primer sequences rather than from the mtDNA itself. c, d) Among the retained reads the proportion of reference and alternative alleles is measured and provides a more unbiased estimate of the level of heteroplasmy.

As the removal of primer-derived sequences by trimming was not satisfactory we developed an alternative data-processing approach to bypass primers without knowing their exact sequences, by seeking reads that spanned primer sites (‘Overarching Read Enrichment Option’; OREO; Fig. 2). This position-specific approach was applied to variants by designing *in silico* ‘probes’, sequences 10–30 nucleotides in length (mean: 16 nt), covering the SNP of interest and extending outside the amplicon ends, which were identified by the sharp drops in the coverage track. OREO requires reads to match probes exactly along their complete lengths, and therefore only selects reads from the two overlapping amplicons which do not end in primer-derived sequences at a tested position. Variants lying in non-overlapping regions are unaffected by primer bias, and therefore do not require correction by OREO. Using one probe for each allele and stringent base-matching allows the specific enrichment and quantification of overarching reads containing reference or alternative SNP alleles. Applying a stringent filtering method is required to clearly distinguish the alleles at the queried position, but also means that reads with random sequencing errors at sites which the probes recognise are also excluded. To minimise this loss, probe length is kept to the minimum that permits selection of overarching reads, while remaining specific. Each probe in a pair has the same length, so any loss is the same for both alleles. At any queried position the coverage of reads considered for variant calling would drop to the level of only one of the two amplicons, namely the one from which the reads lack primer-derived sequences at this position, and therefore the resulting coverage loss is position- and sample-dependent. Probe sequences and an example of use are given in Table S3, and the OREO Bash script is provided in text format at https://www2.le.ac.uk/departments/genetics/people/jobling/mark.

As an example of significant reference bias removal by OREO, at position 16,234 in sample bak-41 the variant T was detected in only 38% (2966/7824) of raw reads, but after OREO processing this increased to 97% (2909/2998) - technically a SNP under the applied thresholds. Thus, applying OREO with the prototype kit we identified SNPs (i.e. variants in >90% of the reads) at 161 of the 1200 positions amplified, defining a total of 101 distinct and diverse mtDNA haplotypes. Prediction of haplogroups from sequences confirmed that the sample set includes most major clades of the mtDNA phylogeny (Fig. S1). Since the samples are not representative of populations, we do not use classical population statistics to analyse our results.

### 3.2 Validation of PowerSeq MPS mtDNA data

We validated the sequences derived following OREO adjustment for 65 samples for which previous data were available, considering base substitutions lying outside the homopolymeric tracts or the AC-repeat region. Discrepancies were observed in 5/65 samples (Table S4), and in all cases these were variants observed in our data that were absent from a reference data source (i.e., potential false positives). For two of these samples, comparative data were available from two sources [23,24], one of which agreed with our data. For the remaining three samples (involving six variant sites in total) the discrepancies cannot be resolved. In conclusion, conservatively treating these six sites as errors in our data, this would yield a false-positive rate of 6/480 = 1.25%; however, we also note that coverage over the discrepant sites in our data is much higher, even after using OREO to select overarching reads when variants lie in overlapping segments (Table S4). No base substitutions observed in the comparative datasets were missed in our data.

### 3.3 Performance of mtDNA amplification across the phylogeny

As previously noted, our diverse sample set (Fig. S1) is suitable for testing the efficiency of MPS multiplexing in a wide variety of control region sequences. SNPs within primer-binding sites can affect PCR efficiency for individual amplicons, and thus the confidence of variant calling. This could be detected by observing particularly low coverage for an amplicon that is generally well covered in the dataset. As an example, the non-overlapping segment of amplicon #3 (Fig. 1, Fig. S3; positions 16,248–16,363) in sample tur-16 showed a mean coverage of 159 ×, compared with a mean of 2207 × for the same segment in other samples at the same multiplexing level, and compared with the mean coverage of 2046 × for other segments within the same sample. This sample belongs to haplogroup HV2a1, and carries two SNPs (16214T and 16217C), which potentially underlie a primer-binding site. This illustrates the fact that sequence variants can affect amplification efficiency for specific amplicons. However, this example involves only one of the 1010 designed amplicons generated from our sample set, decreasing, but not eliminating amplification of mtDNA as template in this region, and therefore the overall performance of the kit in diverse mtDNAs is robust.

### 3.4 Detection and quantitation of heteroplasmy

Given the issues with reference sequence bias and the difficulty of trimming primer-derived sequences from reads as described above, we were interested to ask if the single-reaction multiplex was able to reliably detect and quantify heteroplasmy. For apparent heteroplasmic sites, mixture between different samples could provide a trivial explanation; to address this, we exploited the fact that we used in this study the prototype multiplex kit PowerSeq™ Auto/Mito/Y System (Promega) [12], allowing us to detect evidence of mixtures through examination of autosomal and Y-STR profiles: in the samples with confirmed heteroplasmies, we observed no unexpected STR alleles that could indicate contamination down to a 1% level of the reads (data not shown).

After variant calling and data processing via OREO, we analysed potentially heteroplasmic sites at which both reference and alternative variants were present. Traditionally, based on Sanger sequencing, the levels of heteroplasmy at such sites are described by the minor allele frequency (MAF; ≤50%) down to a technology-dependent threshold below which heteroplasmy cannot be reliably identified. Here, however, since MPS relies on a reference sequence mapping approach, we identified variants based on alternative allele frequency in which an alternative variant is observed in 10–90% of the reads at a site (corresponding to 10% MAF threshold). When an alternative variant at a given site is present in <10% or >90% of the reads, the site is called as homoplasmic (reference or alternative allele, respectively). The 10% MAF threshold represents a similar resolution of heteroplasmy to that of the classical Sanger method [31].

**Fig. 3:**
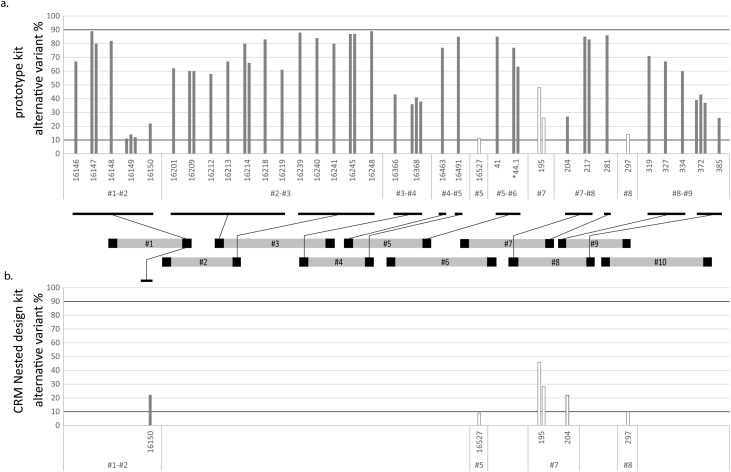
Distribution of apparent heteroplasmies across the control region. Percentages of heteroplasmy (as alternative variant percentage, from 10 to 90%) are shown in the bar charts for each apparently heteroplasmic variant after OREO processing, and arranged by position along the control region. Positions in amplicons are indicated, using the nomenclature of Fig. 1. The asterisk indicates a site displaying single-base indels - all others are base substitutions. Black horizontal bars underneath positions indicate probable primer sequences, also represented as black boxes on the schematic amplicons in the centre of the Figure. White bars in the bar charts indicate positions outside probable primer regions. a) 47 apparent heteroplasmies detected using the prototype kit; note that only four of these survive following our conservative criteria of taking a 10% threshold, and not calling sites under potential primer positions; b) Six heteroplasmies detected using the CRM Nested kit; note that the variant at position 16,150 lies under a primer site, and that the variant at position 204 lies under a primer-binding site in the prototype, but not in the CRM Nested kit design.

After accounting for the primer-derived sequences at the end of the reads we identified 45 apparent heteroplasmic substitutions, at which the minor allele is either the reference or the alternative variant. Such sites were observed in 39 samples at 33 positions, and each of these samples contained between one and three variants. We also identified two samples containing the same single-nucleotide insertion (44.1C) in a heteroplasmic state (showing alternative variant proportions of 69% and 77% in overarching reads). To investigate the status of these apparent heteroplasmic sites, we considered their locations within the control region (Fig. 3a). This shows clearly that these sites cluster in likely primer-binding sites: thus, despite our efforts to remove reference bias by enriching for overarching reads, some bias remains. Our approach filters out reads containing likely primer sequences at their ends, so the remaining bias must be due to primer sequences that are internal to the reads, most likely via overlap extension [32] - the annealing of overlapping single-stranded PCR products, which can prime synthesis from the 3´ end of an already incorporated primer-derived sequence (Fig. S4a). Therefore, heteroplasmic calls are not possible in likely primer binding sites even at a conservative level of 10%. Applying these conservative criteria reduces (at the risk of losing some genuine heteroplasmy) the number of apparent heteroplasmic calls from a total of 47 to four (Fig. 3a). Of these, two are at position 195, a well-known heteroplasmy-prone site [31]. As well as single-nucleotide variants, simple-sequence regions (e.g. homopolymer tracts) were also analysed, including quantitation of length heteroplasmy. We found 284 instances of variants within these regions. Each sample contained between one and four such variants (Table S5).

To assess the potential ability of other readily available approaches to mitigate the effect of primer-derived sequences in variant and heteroplasmy detection we also tested two tools designed for the analysis of mtDNA MPS data. The online tool mtDNA-Server [19] acknowledges the problem with overlapping amplicons and heteroplasmy detection, but does not offer an option to correct for the presence of primers in reads. It gave a very high number of heteroplasmic calls, comparable to our data prior to correction with OREO. The trial version of the commercial software GeneMarkerHTS v1.2.2.1338 (SoftGenetics, LLC) contains the option to input amplicon coordinates, but it is unclear how this information affects data processing; when comparing outputs with and without this option, both gave comparably high numbers of heteroplasmic calls, as in our uncorrected data. Since neither of these tools were aiming to correct for the primer-derived bias that we demonstrated here, OREO is still the most successful tool for decreasing, if not completely removing, bias from the data.

### 3.5 Improved heteroplasmy detection with the CRM Nested System

The improved format of the redesigned PowerSeq™ CRM Nested System multiplexes only mitochondrial amplicons, streamlines the library preparation process and minimises sample loss by using a nested amplification protocol with a single-step PCR including both amplification and incorporation of indexed sequencing adapters.

A subset of 57 samples that showed potential heteroplasmy with the prototype was analysed using the CRM Nested kit to assess how this improved approach affects the accuracy of detection of heteroplasmic sites. The CRM Nested design not only provides potentially higher coverage per sample, but also has the advantage of preventing the internalisation of the primer sequences (compare Fig. S4a and b) because overlapping extension from the single-stranded short amplicons is now prevented by the flanking adapter sequences. We re-examined the 47 heteroplasmic calls observed using the prototype kit; the CRM design resolved nine of these calls as not heteroplasmic (3 reference and 6 alternative alleles; Table S6) even in the raw data, highlighting the effect of previous primer internalisation by overlap extension.

While samples were subject to bead-based size selection during library preparation as per the manufacturer’s protocol, nevertheless a large proportion of the short (52 – 89-bp) amplicons survived the process and therefore further contributed to the non-uniform coverage. Since the new design generates only mitochondrial amplicons, it gives a better defined read-length profile compared to the prototype kit (Fig. S5), which contained amplicons from variable-length STRs as well. This makes it possible to apply *in silico* size selection on the generated data to minimise the effect of potentially interfering short amplicons. After careful examination of the read-length profile plot for each sample generated by FastQC software [33] we defined a cut-off of 95 bp to remove reads generated from these shorter amplicons (green reads in Fig. 2a) using Trimmomatic v0.32 software [22]. The generation of these short amplicons is not uniform among overlapping segments, and the removal of these mostly affects the regions of overlap at positions 16,533-52, 16,363–16509, 109–180 and 16,450–16,509 (Fig. S6), where they are shown to significantly contribute to the coverage observed in these regions. After removing the short amplicons, the remaining 38 calls remained apparently heteroplasmic, although some of the allele proportions changed (Table S6).

Following the removal of the short reads, we applied OREO as an alternative to primer trimming to bypass primer sites and select only overarching reads to call variants. Applying OREO clarified all the 47 heteroplasmic calls as follows: six sites were shown to present clear heteroplasmy, one of which (16150T at 22% in sample hun-5) was successfully identified despite lying under a primer site (Fig. 3b and Table S6); the remaining 41 occurrences were resolved either as reference (three) or alternative variant (38) alleles. These 38 SNP calls clearly demonstrate the high proportion of reference bias conveyed by the presence of primer sequences in the reads, and the importance of accounting for their presence in the dataset. Comparisons of the effects of the different kits and data processing steps on the 47 initially flagged potential heteroplasmic calls after applying OREO are detailed in Table S6.

The nested approach of the CRM kit prevented the internalisation of primers which had been identified as the likely source of the retained reference bias observed in the prototype. This suggested that the direct trimming of primers from read ends would be more effective with the CRM design; however, if the trimmed length is too short, primer bias will be retained, while if it too long, coverage gaps will be introduced. In our experiment, we observe biased SNPs up to 27 bp from the ends of amplicons, suggesting that at least 27 nt should be trimmed from reads to remove primer bias. However, such trimming creates a small gap in the overlap region of amplicon #2 and #3 covering variable positions 16,222 and 16,223, thus introducing false negative calls. Overall, although the conventional trimming approach has the virtue of simplicity, this comes at the cost of introducing coverage gaps, a problem that does not apply to OREO.

Observing the effectiveness of the CRM kit design when followed by appropriate data processing steps (*in silico* size selection and OREO, Table S6, Figure S7), and permitted by the higher coverage obtained, we lowered our heteroplasmy calling threshold from 10% to 5% (Figure S2). Applying the 5% threshold flagged 102 sites which were compared between the two kits after data processing, and are shown in Table S7; this identified a further six heteroplasmic sites.

In total, we detected twelve heteroplasmic sites with alternative variant proportion between 5 and 95%. These lie at eleven different positions of the control region (182, 195, 204, 207, 297, 316, 16,069, 16,093, 16,129, 16,150 and 16,527) in twelve samples, and range from 8 to 95% alternative variant proportion. Several of these are often reported as heteroplasmic [9,43]. For example, position 16,093 in the adjusted CRM data was observed once above (and also twice below) the 5% reporting threshold. Only two of these are transversions (297C 9% and 316C 16%), both positioned away from primer-binding sites (details in Table S7). After applying OREO, the number of homoplasmic variants identified in the control region increased to 197, including five simple indels.

### 3.6 Detection of numt sequences

In a multiplex control-region assay, reduced coverage for a specific amplicon [7] could permit the detection of a numt sequence, as a pattern of variants resembling multiple closely linked heteroplasmic sites (Fig. S3). In the low-coverage interval of the sample (tur-16) described in the prototype data above (see: *Performance of mtDNA amplification across the phylogeny*), fourteen sites in a window of 114 bp showed mixed allele calls with the minor component at a level of ˜28%. The minor-component sequence was used in a blastn query against the nucleotide collections of GenBank, and returned a known polymorphic numt sequence inserted after chr11:49,862,017 (GRCh38) on chromosome 11p11.12, first described by Zischler et al. in 1995 [34], and subsequently noted by others [[35], [36], [37], [38], [39]]. Given this knowledge, we surveyed numt-specific variants in the sequence flanking the 114-bp window and observed the same level of numt-specific reads at three additional sites underlying a much higher coverage of cognate mtDNA reads (Fig. S3).

We identified this numt sequence because of the low-coverage amplicon in tur-16, but having discovered it, asked whether it also existed as a low-level component of reads in other samples. Similarly low-levels of this sequence were detected (at a mean of 0.67% of the reads) in an additional 43 samples of diverse continental origins, showing that the numt sequence may be polymorphic, and present at high frequency in our diverse sample. This is consistent with previous findings of the geographical distribution of this insertion [35,37], and with the frequency in the 2504 samples of the 1000 Genomes Project (where it is annotated as the structural variant esv3626324 in dbVar, and also as the SNPs rs116522696 and rs115254439). We inspected the 29 of our samples that were also included in the 1000 Genomes Project: in 1000 Genomes data, 9/29 samples lack the insertion, and our data agree with these. However, the numt sequence is known to be present in all 20 remaining samples, but we detect it in only four, indicating a high false-negative rate.

We also detected this numt sequence in two-thirds of the samples processed with the PowerSeq™ CRM Nested System, and while again no false positives were detected compared to 1000 Genomes Project data, detection of the presence or absence of the numt sequence between the two kit types was not always consistent.

## 4 Discussion

Here, we have analysed a set of 101 diverse human DNA samples to investigate the calling of variants in the mitochondrial control region using multiplex MPS-based approaches.

Coverage across the control region is non-uniform in overlapping amplicon approaches (Fig. 1, S6). Excess coverage may result partly from differences in amplification efficiency among amplicons, but also, thanks to the single-reaction kit design, from the preferential generation of shorter amplicons which occupy substantial sequencing capacity of an MPS run (Fig. 2). Sequence reads from shorter products contain a high proportion of primer-derived sequences which cannot be precisely removed by conventional trimming because the primers are proprietary, and which introduce reference sequence bias in the data. This led us to develop an alternative data-processing approach (Overarching Read Enrichment Option, OREO), to bypass the bias introduced by primers at the ends of reads by retaining only overarching reads.

Following application of OREO, analysis of the mtDNA control region in a set of 101 samples using the prototype kit design (PowerSeq™ Auto/Mito/Y System) showed generally robust amplification and sequencing, despite the high diversity of analysed mitochondrial genomes, which covers a wide range of the mtDNA phylogeny (Fig. S1). All 101 samples presented different sequences, defined by variation among 161 SNPs within the control region. Validation against independent data from whole mtDNA sequences for 65/101 samples [23,24] showed a high degree of concordance with no false negative variants. We estimated a conservative false-positive rate of 1.25%, but given the high sequence coverage of our data over the relevant sites we are confident that the error lies in the comparative data rather than our own, and believe that the false-positive rate is in reality lower than this.

Heteroplasmy is a ubiquitous phenomenon, and needs to be considered in any forensic analysis of mtDNA [4]. As with the detection of numt sequences, the introduction of MPS has improved the sensitivity of detecting lower-level heteroplasmy compared to Sanger-based methods [40]. Application of OREO, an alternative to primer trimming, reduced the number of potential heteroplasmic variants by removing the bias introduced by primer sequences at the ends of reads, but not those that are internalised by overlap extension (Fig. S4).

The PowerSeq™ CRM Nested design prevents overlap extension by adding adapter sequences onto the ends of the amplicons. Data from this kit can then be processed to remove reads from the short amplicons by *in silico* size selection, and further improved by applying OREO when calling variants and quantifying heteroplasmies at primer sites. The combination of improved chemistry of the CRM kit and appropriate data processing with OREO allowed us to consider the whole control region to accurately call variants and heteroplasmies down to the level of 5%, identifying 197 different variants and twelve point heteroplasmies in this sample set. We note that other MPS-based approaches permit lower thresholds, thanks to different kit designs or deep sequencing [41,42]; however, for the general purposes of variant and heteroplasmy detection our limit of 5% seems sufficient.

Considering kit design more generally, a non-overlapping two primer-mix option (following the original approach [7]) remains preferable to a single-reaction multiplex. Even though OREO can provide an alternative to primer trimming and can overcome the artefacts associated with the single-reaction approach, the short amplicons of overlapping regions can take up significant capacity on the surface of the sequencing chip. While this is manageable when processing ideal reference quality samples, as we demonstrated here, the effect will be more detrimental when sample quality is non-ideal: preferentially amplified short amplicons will be further elevated, and longer amplicons reduced, enhancing the bias present at the primer sites in variant calls.

The human genome is well known to contain many numt sequences [43,44] and some of these are polymorphic insertions which can be used in human population studies and phylogenetic analyses [37,38]. These divergent copies of mtDNA fragments are not expected to cause particular concern in short-amplicon sequencing of the control region, due to the multiple copies of cognate mtDNA template, and the extremely high coverage of the resulting cognate reads. Indeed, co-amplified numt sequences are generally below the detection limit of conventional Sanger sequencing-based assays, although they have been detected with denaturing gradient-gel electrophoresis, which was intended to resolve lower level heteroplasmies [45]. The depth and precision of MPS can allow the detection of low-level numt-derived reads [39], but again these reads do not normally reach the variant calling threshold and are therefore not reported.

In our study, however, reduced amplification efficiency for one amplicon in one individual, due to the presence of SNPs within a primer-binding site, allowed a numt sequence to be detected (Fig. S3). This prompted a wider screen for the same numt sequence, and we found it to be detectable with both kit types at very low levels in more than half of our sample set (though comparisons to the 1000 Genomes Project data show a high false-negative rate, so its true frequency must be higher). Considering that these nuclear templates are neither specifically targeted nor enriched for detection, but observed only as a by-product overshadowed by usually overwhelming cognate mitochondrial templates, it is not surprising that they are observed inconsistently. Relevant variables include the individual underlying variants in the sample, the relative amplification success of the nuclear and mitochondrial templates, the multiplexing level and other run parameters affecting the coverage of the sample. Thus, despite the high population frequency and widespread distribution of this polymorphic numt sequence [35,37], it interferes with variant calling in reference samples only rarely. In non-forensic applications numt sequences could interfere with correct reporting of mitochondrial DNA variants [46]. However, in the analysis of casework samples where low-level minor components are of particular interest [39], numt sequences could become visible, requiring removal [47], or may be falsely interpreted as heteroplasmies or mixtures.

## 5 Conclusion

We have shown that single-reaction multiplexes provide robust tools to analyse mtDNA control region variation in diverse samples. The PowerSeq™ CRM Nested System, provided appropriate data processing is used to mitigate reference sequence bias, can identify variants in the whole control region and is able to measure heteroplasmies down to a 5% threshold (outperforming Sanger sequencing). However, where sample availability allows, a two-reaction mtDNA control region system, in which reference sequence bias is absent, may be preferable. All commercial kits (whether forensic or not) which use overlapping amplicons suffer from the conflicting needs of the manufacturer to protect proprietary primer information, and the user to know their sequences to enable appropriate data analyses. Our OREO approach, as an alternative to primer trimming, allows users to bypass even unknown primer sequences, and to eliminate the bias conveyed by the presence of primers in overlapping amplicon sequencing studies.

## Conflicts of interest

None.
