# Supplementary material for: Mitigating the effects of reference sequence bias in single-multiplex massively parallel sequencing of the mitochondrial DNA control region
Source: Forensic Sci Int Genet. 2019 May;40:9–17. doi: 10.1016/j.fsigen.2019.01.008 (PMC6461131; doi:10.1016/j.fsigen.2019.01.008)
Supplement: Supplementary file 1 [file mmc1.pdf]

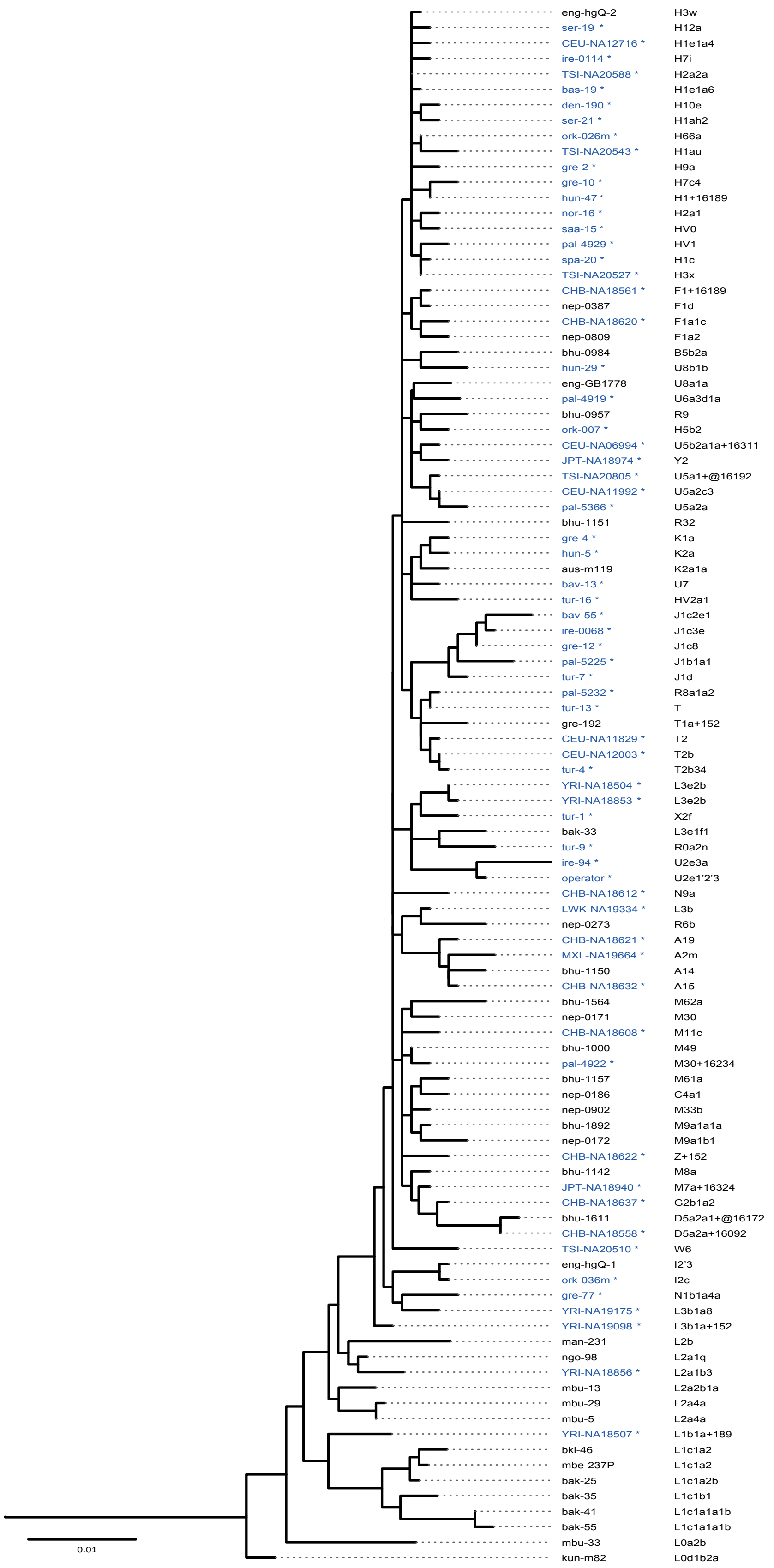

Figure S1. Maximum Likelihood phylogenetic tree of the mitochondrial control region sequences (from position 15989 to 619) obtained from 101 samples

The tree was inferred by using the Maximum Likelihood method based on the Jukes-Cantor model [1] using MEGA6 [2] and edited by FigTree v.1.4.3 [3]. Branch lengths measured in the number of substitutions per site. The tree was rooted to a *Pan troglodytes* mitochondrial sequence (NC_001643.1), not shown here. Sample names are noted at the tips with haplogroups on the right as predicted by HaploGrep2 [4]. Samples validated against reference datasets are in blue font and marked with an asterisk.

1. Jukes and Cantor (1969). Evolution of protein molecules. In Munro HN, editor, Mammalian Protein Metabolism, pp. 21-132, Academic Press, New York.
2. Tamura et al. (2013). MEGA6: Molecular Evolutionary Genetics Analysis version 6.0. Molecular Biology and Evolution 30: 2725-2729.
3. Rambaut, A. (2014). FigTree v1.4.3. Available at: http://tree.bio.ed.ac.uk/software/figtree/
4. Weissensteiner et al. (2016). HaploGrep 2: mitochondrial haplogroup classification in the era of high-throughput sequencing Nucleic Acids Research.8;44(W1):W58-63.

PROTOTYPE

error bins	positions within error bin
<0.5%	1000
0.5-1%	91
1-2%	52
2-3%	11
3-4%	8
4-5%	6
5-10%	11
>10%	21

required min coverage values			bases below threshold		samples affected	
homoplasmic call	heteroplasmy major/ minor	threshold	#	%	#	%
20	4000+/20					
20	4000/20	**0.50%**	50258	41.47%	99	98.02%
20	2000/20	**1%**	16306	13.45%	91	90.10%
20	1000/20	**2%**	3019	2.49%	39	38.61%
20	667/20	**3%**	1328	1.10%	27	26.73%
20	500/20	**4%**	783	0.65%	20	19.80%
20	400/20	**5%**	636	0.52%	16	15.84%
20	200/20	**10%**	380	0.31%	8	7.92%

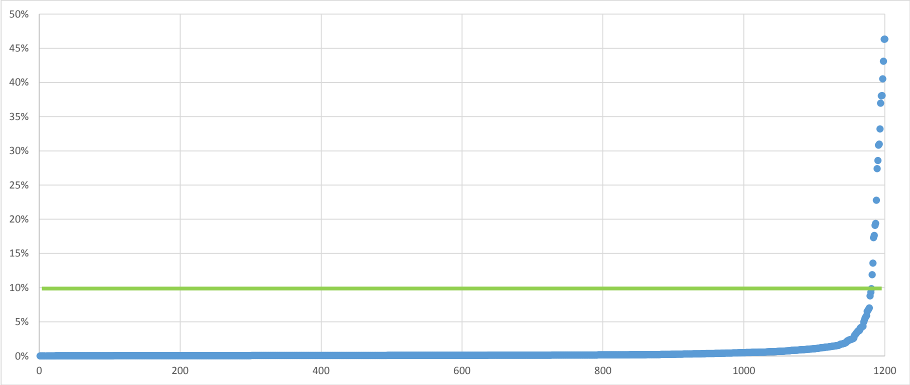

Prototype, mtDNA-based substitution error rates	
A%	6.86E-02(±4.24E-01)
C%	1.99E-01(±1.42E+00)
G%	4.35E-02 (±6.34E-01)
T%	5.95E-02 (±5.55E-01)
Overall%	3.70E-01 (±8.33E-01)

Prototype, Amelogenin-based substitution error rates	
A%	3.75E-03 (±6.58E-03)
C%	1.49E-02 (±2.40E-02)
G%	7.41E-03 (±1.89E-02)
T%	5.73E-03 (±8.25E-03)
Overall%	3.18E-02 (±1.71E-02)

CRM NESTED

error bins	positions with error bin
<0.5%	986
0.5-1%	110
1-2%	40
2-3%	21
3-4%	9
4-5%	3
5-10%	12
>10%	19

required min coverage values			bases below threshold		samples affected	
homoplasmic call	heteroplasmy major/ minor	threshold	#	%	#	%
20	4000+/20					
20	4000/20	**0.50%**	2110	1.74%	39	68.42%
20	2000/20	**1%**	879	0.73%	25	43.86%
20	1000/20	**2%**	373	0.31%	12	21.05%
20	667/20	**3%**	288	0.24%	8	14.04%
20	500/20	**4%**	58	0.05%	6	10.53%
20	400/20	**5%**	37	0.03%	6	10.53%
20	200/20	**10%**	14	0.01%	4	7.02%

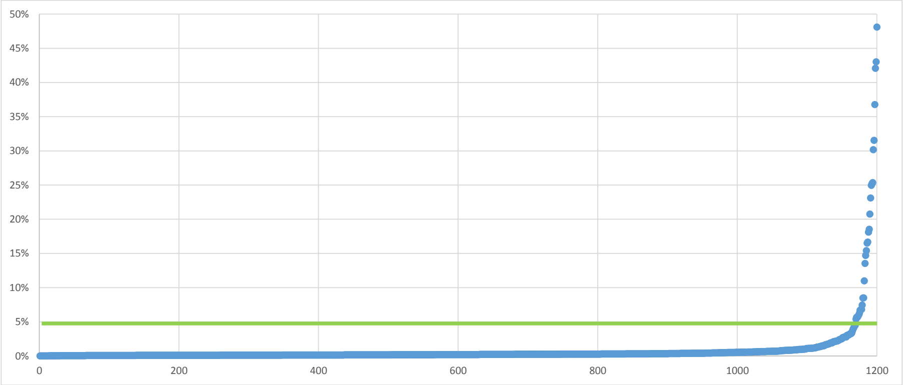

CRM Nested, mtDNA-based substitution error rates	
A%	7.35E-02(±7.02E-01)
C%	2.43E-01(±1.35E+00)
G%	8.17E-02 (±3.79E-01)
T%	6.93E-02 (±5.80E-01)
Overall%	4.68E-01(±8.09E-01)

Figure S2. Setting reporting thresholds and calculating substitution error rates
Conservative substitution error calculations for the prototype and the CRM kits based on mtDNA reads for all samples were carried out as per Rathbun et al. (2017)[1]. The top left table for each kit type shows the number of positions that are affected by each error bin. Conservative designation means that any non-major variants (even true heteroplasmies) are included in the calculation.
The top right table for each kit type shows the sliding scale requirements for coverage corresponding to each potential heteroplasmy detection threshold, the consequent loss of bases not covered to this level, and the proportion of samples affected by this loss. The best compromise of these requirements was set to be used as reporting thresholds and is highlighted in green.
The graph for each kit shows the distribution of error rates per position amplified, in increasing order, with the green horizontal line representing the set thresholds, also demonstrating that our thresholds are set way above the background substitution error levels.
The bottom tables summarise the details of these substitution error rates based on the mtDNA reads. For the prototype, these were also calculated for the amelogenin reads, taking advantage of this co-amplified marker, which is not affected by stutter or heteroplasmy, and therefore ideal to define the template-type-independent technical rate of substitution errors.

1. Rathbun et al. (2017). Considering DNA damage when interpreting mtDNA heteroplasmy in deep sequencing data. Forensic Sci Int Genet. 2017;26:1-11.

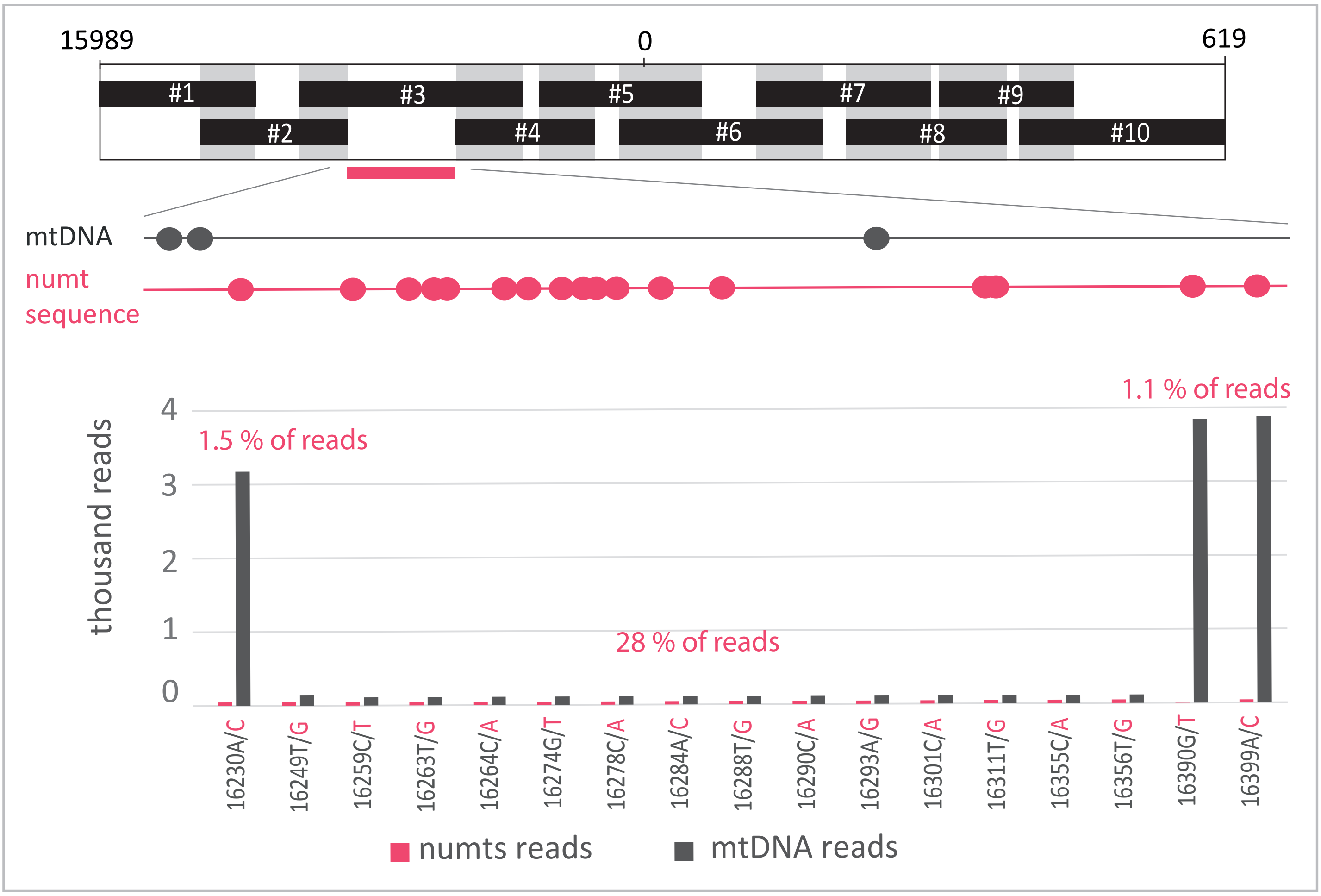

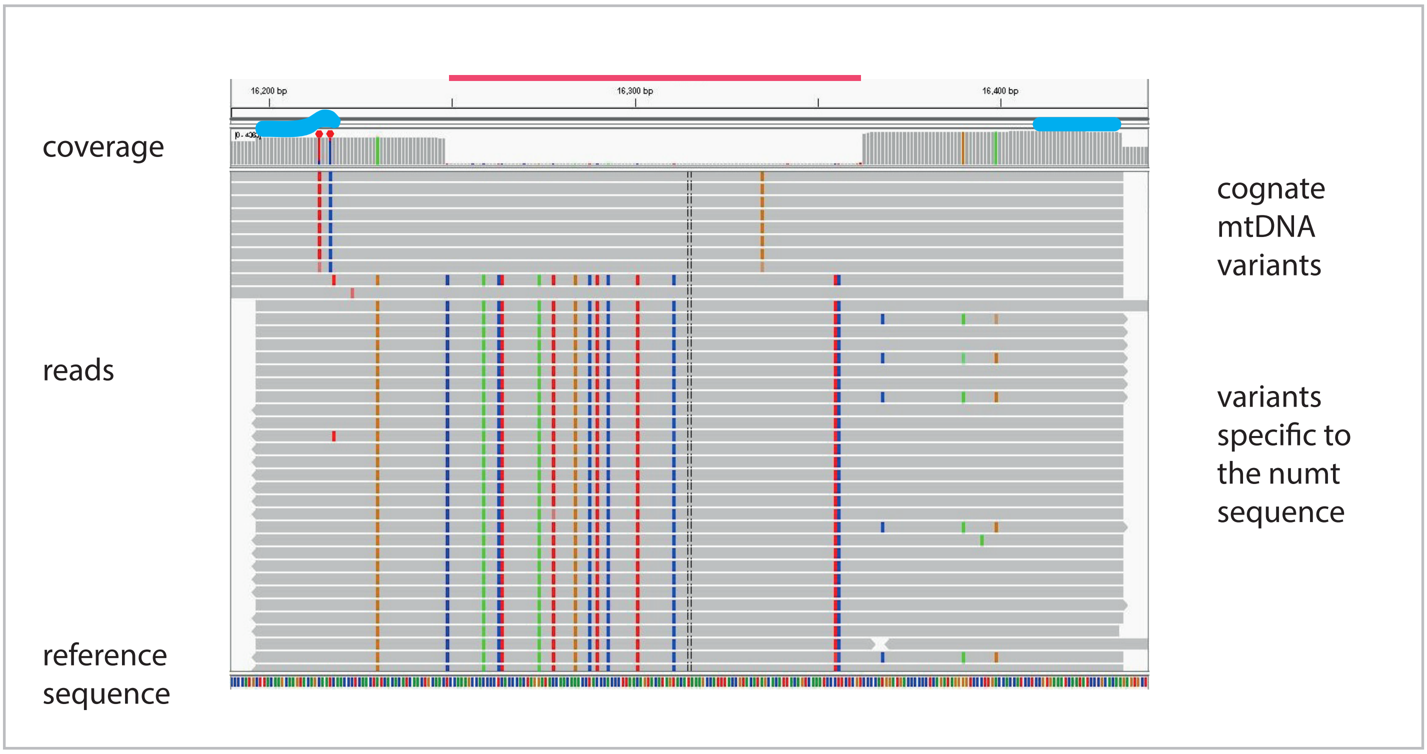

Figure S3. Low coverage of amplicon #3 and detection of the numt sequence in sample tur-16
In the top panel a schematic diagram showing the detection window (crimson bar) of the numt sequence in relation to the ten overlapping amplicons and a corresponding cartoon indicating the haplotypes of variants observed in the mtDNA (grey) and the numt sequence (crimson). At the bottom panel an annotated IGV screenshot demonstrates the approximate sites of the primers (blue rods) binding and highlights where the variants (16214T and 16217C; red dots) affect efficient binding. The top track shows coverage of reads of the region, while a portion of reads are shown in the track below. Bottom track shows reference sequence.

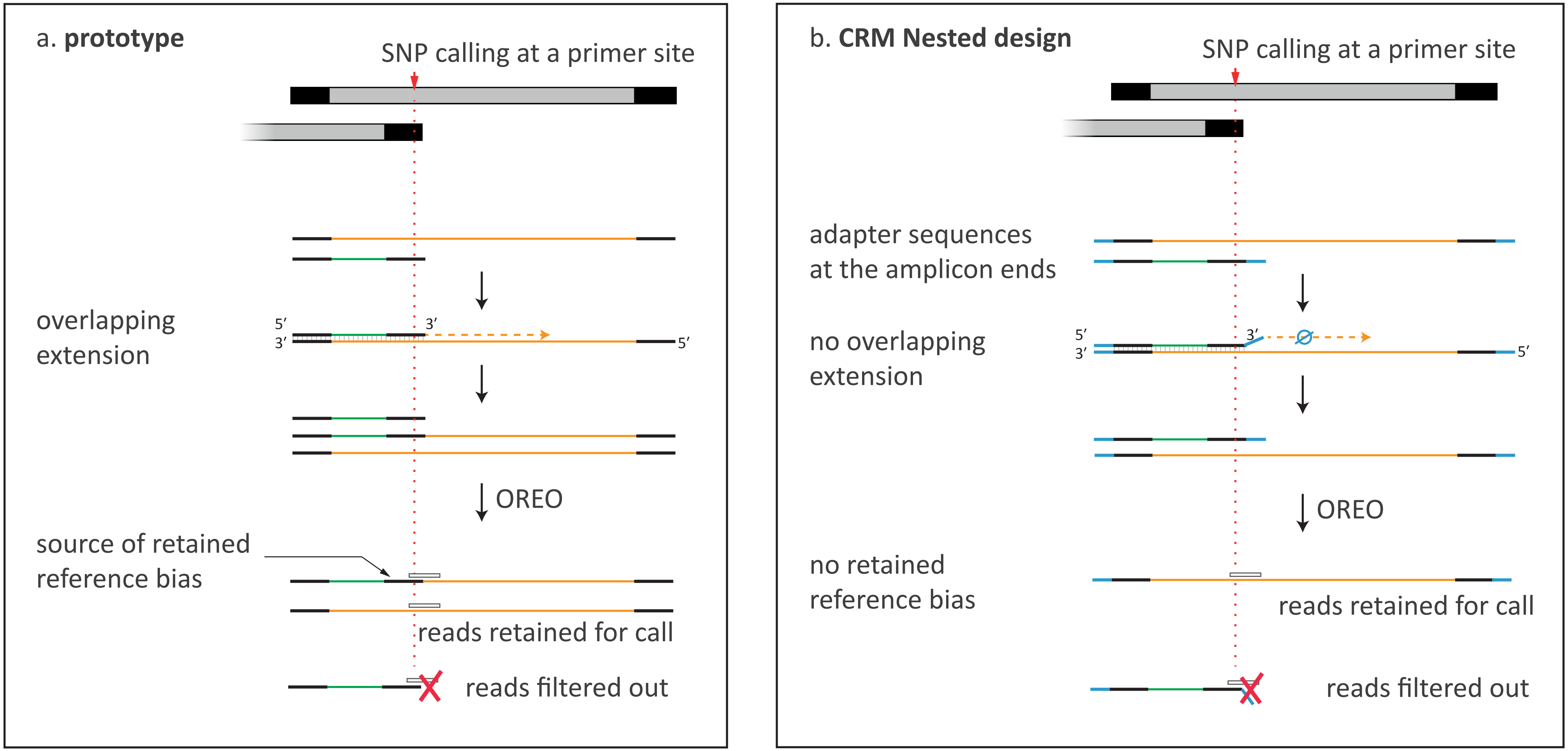

Figure S4.
Annealing and extension of overlapping single-stranded PCR products in the prototype kit introduces reference sequence bias internal to the reads. The Nested CRM design prevents extension of overlapping single-stranded PCR products and internalisation of primers.

a) Shorter amplicons (green horizontal bars), similarly to primers (black boxes and black thicker bars), can anneal to the designed amplicons (orange horizontal bars) when single-stranded during amplification and generate products that contain primer-derived sequences internal to the reads. OREO uses probes (white boxes) to filter out reads that do not span over the primer site to reduce reference bias from primers, however the hybrid products of overlapping extension escape this process, and therefore carry over reference bias into the retained reads.
b) In the CRM Nested design the tailing adapters (blue bars) at the end of the amplicons prevent overlapping extension and internalisation of primer-derived sequences. The remaining primer-derived sequences are successfully filtered out by OREO.

PoweSeq™ Auto/Mito/Y System prototype kit read-length profile plot

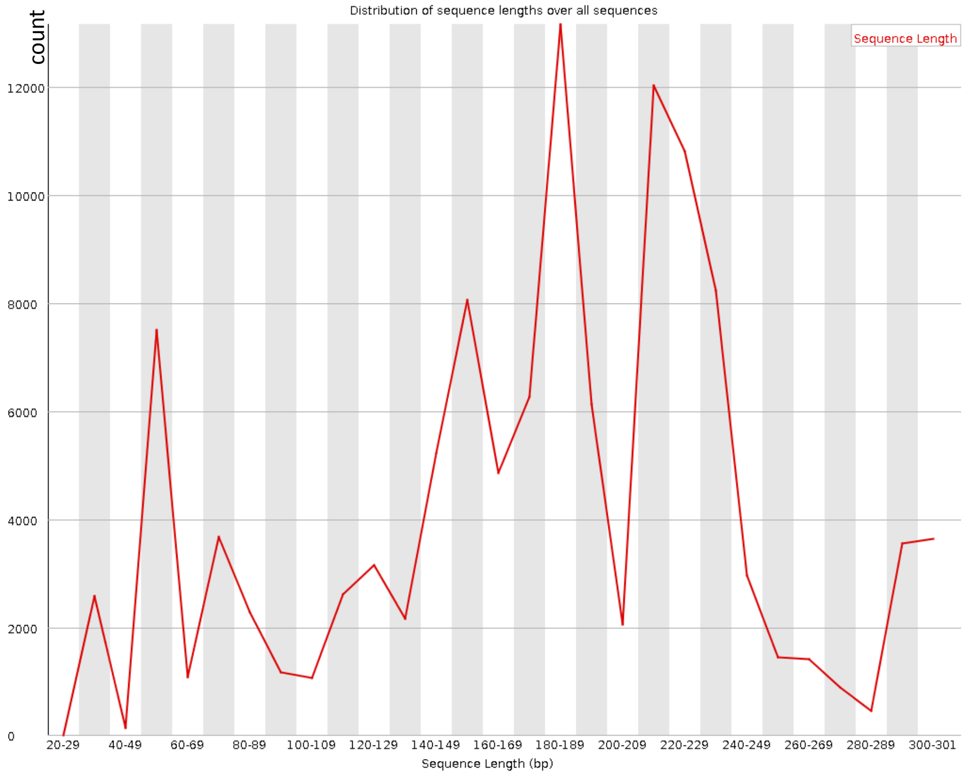

PowerSeq™ CRM Nested System kit read-length profile plot

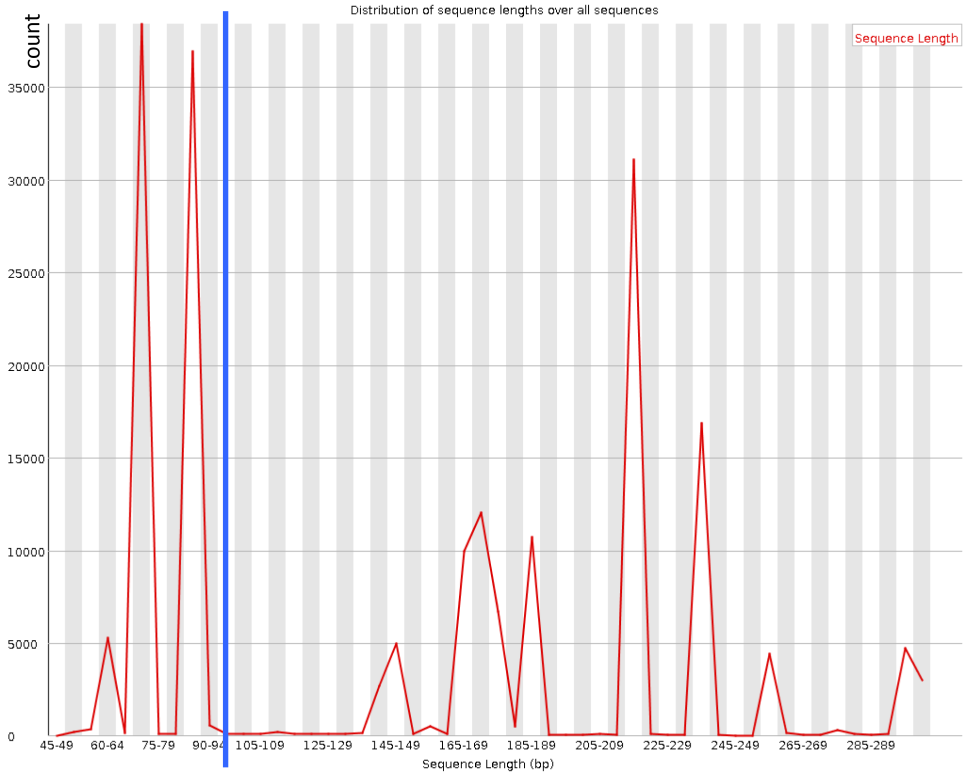

Figure S5. ***In silico*** **size selection to remove reads corresponding to short amplicons in the CRM kit.**

The updated CRM design allows the precise removal of reads generated from the short amplicons from the QC-ed fastq files using bioinformatic trimming tools. Here, after confirming ideal threshold in FastQC[1] software read-length profile function, we used Trimmomatic[2] to remove reads shorter, than 95 bases – represented by the blue vertical line – from sample CHB-NA18608.

1. S. Andrews, FastQC: a quality control tool for high throughput sequence data. (Available online at http://www.bioinformatics.babraham.ac.uk/projects/fastqc/, 2010).
2. A.M. Bolger, M. Lohse, B. Usadel, Trimmomatic: a flexible trimmer for Illumina sequence data, Bioinformatics 30 (2014) 2114-20.

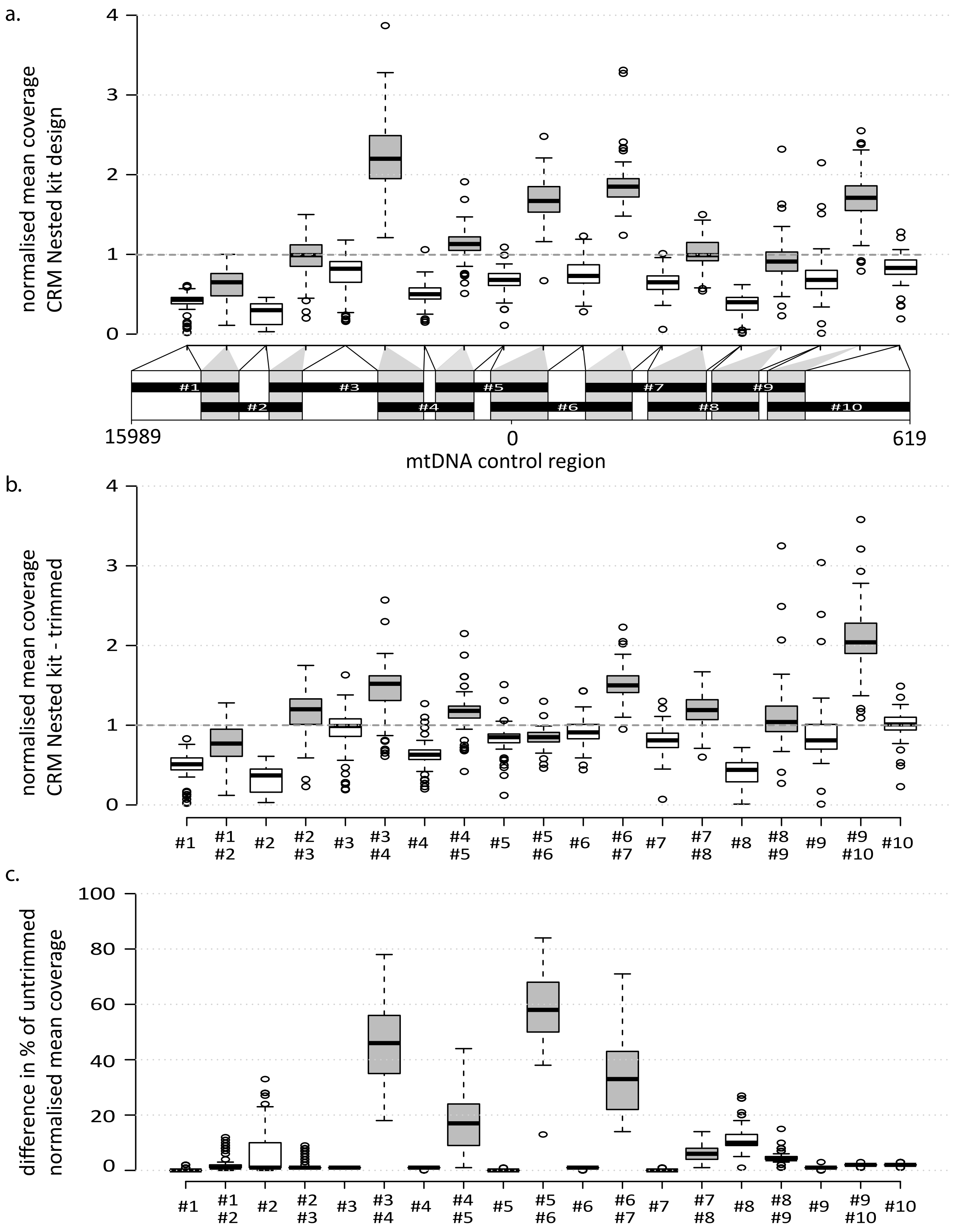

Figure S6: Non-uniform coverage over the control region with the PowerSeq™ CRM Nested kit design.

The figure follows Figure 1. as described there. Part a. shows the normalised mean coverage values for the CRM Nested design before trimming off the short amplicons, b. shows it afterwards. Part c. plots the percentage of trimmed off reads per region, showing short amplicons are formed in a non-uniform fashion.

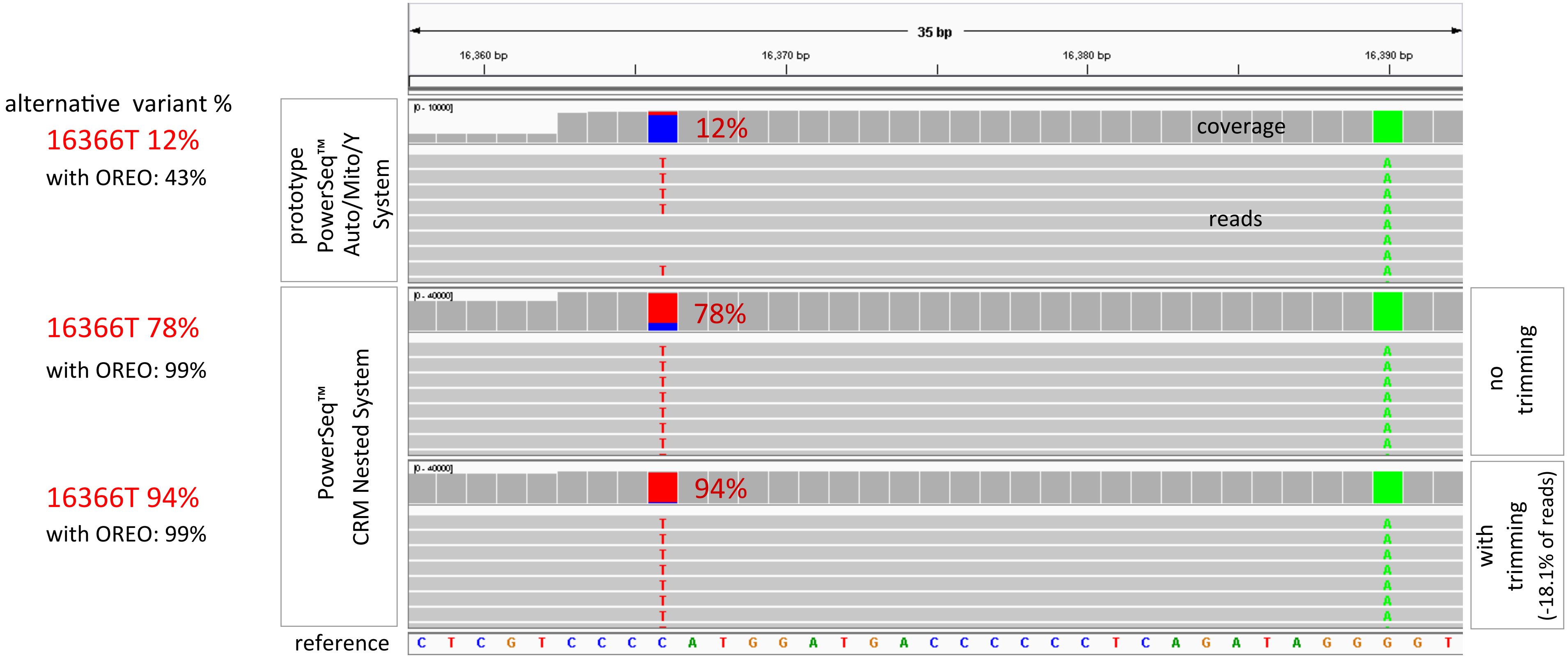

Figure S7. Example of effects of kit design and data processing in mitigating reference sequence bias at an affected variant position.

In sample bav-55 at position 16366 an alternative variant 16366T is observed in different proportions.
This position is three bp away from the amplicon/read end, therefore reasonable to consider it being under a primer site. Variant 16390A is clearly not affected, as being further away from the amplicon/read end, thus not showing primer-derived bias. Top track shows data from the prototype kit, lower two tracks show data from the new CRM Nested kit, of which bottom track displays data trimmed of short amplicon derived reads. OREO data processing is shown to improve all three data sets.
